# Observations on Medical Studies

**Published:** 1813-04

**Authors:** 


*To the Editors of the Medical and Physical Journal.*

GENTLEMEN,

IN the younger branches of the faculty, there are many who, from unavoidable disadvantages, have not the means of acquiring a necessary knowledge, previous to attending the London hospitals and lectures. Most masters want either the opportunity or inclination to instruct their apprentices, and are altogether indifferent to their improvement, provided they attend to the business. To this neglect, which tends to instil an idea of self-sufficiency in the minds of young men, we must in a great measure impute the extreme ignorance of many. For myself, and others who labor under such unpleasant circumstances, I beg leave to solicit, that some of your learned correspondents would, in an early Number of your valuable Journal, favor us with a list of the best books, theoretical and practical, on all the sciences in or relating to the medical art; with their opinions, where a student ought to commence, and how to conduct his studies with the most advantage, in order to acquire a general knowledge of the whole. Trusting to your avowed desire of aiding your art, I hope that you will insert the above, and that some gentleman adequate to the subject will condescend to favor us, through your medium, with his observations upon it; which will confer a lasting obligation on young students in medicine, among whom

I have the honor to be, Gentlemen,
Your obedient Servant,
*Yorkshire*, JUVENIS.
*March* 4, 1813.

